# Efficacy and Safety of Guselkumab in Real-World Evidence: A Systematic Review and Meta-Analysis

**DOI:** 10.3390/jcm15103692

**Published:** 2026-05-11

**Authors:** Jose Manuel Dodero-Anillo, Marta Fernandez-Pujol-Marzo, Maria Jose Pedrosa Martinez, Ricardo Ruiz-Villaverde, Jose Carlos Armario-Hita

**Affiliations:** 1 Clinical Pharmacology, Hospital Universitario Puerto Real, 11510 Cadiz, Spain; 2 Pharmacology Department, Faculty of Medicine, University of Cadiz, 11002 Cadiz, Spain; 3 Dermatology and Venereology, Hospital Universitario San Cecilio, Instituto Biosanitario de Granada, 18016 Granada, Spain; 4 Dermatology and Venereology, Hospital Universitario Puerto Real, 11510 Cadiz, Spain

**Keywords:** guselkumab, psoriasis, psoriatic arthritis, real-world evidence, systematic review, meta-analysis, IL-23, PASI, DAPSA

## Abstract

**Background:** Guselkumab, a selective anti-IL-23p19 monoclonal antibody, has shown high efficacy in randomized trials for moderate-to-severe psoriasis and active psoriatic arthritis (PsA). Real-world evidence is essential to assess treatment performance in broader and more heterogeneous patient populations. **Objectives:** To synthesize the available real-world evidence on the effectiveness and safety of guselkumab in psoriasis and PsA, and to quantify pooled clinical responses across clinically relevant follow-up windows. **Methods:** A systematic review and meta-analysis was conducted according to PRISMA 2020. PubMed/MEDLINE, MEDLINE, and Web of Science were searched for observational real-world studies of guselkumab in adults with psoriasis and/or PsA. Primary pooled outcomes were PASI 90 and PASI 100 in psoriasis and DAPSA < 14 in PsA. Random-effects meta-analysis of proportions was performed using a logit transformation. Cohort overlap was explicitly assessed and overlapping publications were not allowed to contribute concurrently to the same pooled estimate. **Results:** Thirty-four studies were included (29 psoriasis, 5 PsA). In psoriasis, pooled PASI 90 response rates were 50.8% (95% CI 46.8–54.8) at 12–16 weeks, 68.4% (95% CI 66.3–70.4) at 20–28 weeks, 71.2% (95% CI 64.9–76.8) at 36–60 weeks, and 77.1% (95% CI 74.9–79.3) at ≥96 weeks. Pooled PASI 100 response rates were 49.8% (95% CI 47.5–52.2) at 20–28 weeks and 49.7% (95% CI 47.1–52.3) at 36–60 weeks. In PsA, pooled DAPSA < 14 response rates were 56.9% (95% CI 23.0–85.3) at 20–28 weeks and 69.5% (95% CI 62.5–75.7) at 48–60 weeks. Safety reporting was heterogeneous, but serious adverse events and discontinuations due to adverse events were uncommon. **Conclusions:** Real-world evidence supports guselkumab as an effective treatment for psoriasis and a clinically useful option for PsA, with a safety profile broadly consistent with the known trial experience. Interpretation remains limited by observational designs, heterogeneous denominators, and inconsistent safety reporting.

## 1. Introduction

Psoriasis is a chronic immune-mediated inflammatory disease affecting the skin and, in a substantial subset of patients, the joints and entheses through the clinical spectrum of psoriatic arthritis (PsA). The burden of disease extends well beyond the cutaneous phenotype. Psoriasis is associated with impaired health-related quality of life, work productivity loss, sleep disturbance, stigmatization, and increased cardiometabolic and psychiatric comorbidity, while PsA adds disability, structural damage risk, and a distinct long-term impact on function and quality of life [1,2,3]. From the therapeutic perspective, both diseases exemplify the transition from broad immunosuppression toward pathway-specific biologic intervention. The IL-23/Th17 axis is central to the pathobiology of psoriasis and PsA. IL-23 promotes differentiation, expansion, and maintenance of IL-17-producing T-cell populations, with downstream effects on keratinocyte activation, synovial inflammation, and enthesitis [4]. This mechanistic rationale led to the development of anti-IL-23 therapies, among which guselkumab was the first selective anti-p19 monoclonal antibody to be approved for moderate-to-severe plaque psoriasis and subsequently for active PsA.

Randomized clinical trials have established the efficacy of guselkumab in both conditions. In psoriasis, the phase III VOYAGE 1 and VOYAGE 2 trials demonstrated that guselkumab was superior to placebo and adalimumab for achieving PASI 90 and other clinically meaningful skin outcomes [5,6]. In PsA, the phase III DISCOVER-1 and DISCOVER-2 studies confirmed significant benefit across joint and skin domains, and the COSMOS trial extended these observations to patients with inadequate response to TNF inhibitors [7,8,9]. These trials collectively positioned guselkumab as a high-efficacy option in contemporary treatment algorithms.

However, pivotal trials do not fully capture the complexity of routine care. Patients seen in clinical practice may be older, more comorbid, more heavily pretreated, more obese, or may present with mixed and difficult-to-treat phenotypes, including scalp, palmoplantar, genital, nail, enthesitic, or axial features. Treatment persistence, dosing delays, switching patterns, and variations in follow-up intensity also differ from trial conditions. As a result, real-world evidence (RWE) plays a crucial complementary role by addressing external validity and pragmatic effectiveness.

RWE on guselkumab has grown rapidly over the past several years. Studies from Germany, Italy, Spain, Finland, North America, and other settings have described favorable outcomes in biologic- naive and biologic-experienced patients, including those with relevant comorbidities or difficult-to-treat disease [10,11,12,13,14,15,16,17,18]. In parallel, more recent multicenter cohorts have expanded the evidence base on long-term treatment persistence, maintenance of deep skin clearance, and effectiveness in PsA [16,17,18,19,20,21,22,23,24]. Yet direct interpretation of this literature is challenging. Cohort overlap between sequential publications from the same network or registry is common; outcomes are reported using variable denominators (intention-to-treat, evaluable patients, or completer populations); follow-up schedules are heterogeneous; and adverse-event reporting is often inconsistent.

A further challenge is that the real-world literature on guselkumab spans two related but methodologically distinct conditions. Psoriasis studies preferentially report PASI response thresholds, absolute PASI targets, and patient-reported quality-of-life endpoints such as DLQI. By contrast, PsA cohorts may center on DAPSA, MDA, LEI, pain visual analogue scales, enthesitis indices, and drug retention. Although it is attractive to present both conditions within the same broad review, the two evidence streams should not be pooled indiscriminately.

A key methodological challenge in the available real-world literature is the frequent presence of partially overlapping cohorts across sequential publications, registries, and subgroup analyses. This issue is often insufficiently addressed in evidence syntheses and may lead to artificial inflation of precision if the same underlying populations are counted more than once. In addition, existing real-world reports vary substantially in denominator definitions, follow-up schedules, and outcome reporting, making direct interpretation difficult. The novelty of the present study lies in the explicit analytical resolution of cohort overlap and in the structured quantitative synthesis of clinically relevant outcomes across predefined follow-up windows. Psoriasis and psoriatic arthritis were considered within the same broad review because they share a common IL-23-driven pathogenic axis and are treated with the same targeted biologic agent. However, given their clinical and methodological differences, quantitative synthesis was performed separately for cutaneous and articular outcomes. Against this background, we performed a systematic review and meta-analysis of guselkumab in real-world practice, with separate quantitative syntheses for psoriasis and PsA. Our objectives were to: (i) describe the qualitative landscape of published RWE; (ii) generate pooled estimates for key clinical outcomes across clinically coherent follow-up windows; (iii) evaluate safety reporting in routine practice; and (iv) resolve cohort overlap explicitly so that pooled estimates reflect independent analytical contributions rather than serial reporting from the same populations.

## 2. Materials and Methods

### 2.1. Study Design and Reporting Framework

This study was designed as a systematic review and meta-analysis of real-world studies of guselkumab in psoriasis and was conducted in accordance with the PRISMA 2020 statement and explanation-and-elaboration documents [1,25]. The manuscript was structured according to Frontiers in Pharmacology guidance for Original Research articles, using explicit sections on study selection, data handling, quantitative synthesis, and limitations. The protocol was registered in PROSPERO (ID: CRD420261346065).

### 2.2. Information Sources and Search Strategy

A structured literature search was undertaken in PubMed, MEDLINE, and Web of Science. The search strategy combined controlled vocabulary and free-text terms related to guselkumab, psoriasis, psoriatic arthritis, and real-world practice. Core terms included “guselkumab”, “psoriasis”, “psoriatic arthritis”, “real-world”, “real life”, “observational”, and “non-interventional”. The full search strategy for at least one database (PubMed/MEDLINE) is provided in Supplementary File S1 in accordance with PRISMA 2020 recommendations. The final search strategy was adapted to the syntax of each database and supplemented by backward reference screening of relevant studies and review articles. The electronic search was conducted up to 10 October 2025. No language restrictions were applied during the search stage. Gray literature was not systematically searched.

The original search identified 249 records in total: PubMed (n = 90), MEDLINE (n = 76), and Web of Science (n = 83). After removal of 150 duplicate records, 99 records underwent title and abstract screening. Twenty-seven records were excluded at title level and 24 were excluded after abstract review. Forty-eight full-text articles were assessed in detail, of which 14 were excluded after full-text evaluation, leaving 34 studies for final inclusion (29 psoriasis and 5 PsA). These numbers were carried forward into the final PRISMA flow diagram integrated in the manuscript.

### 2.3. Eligibility Criteria

Eligible studies were observational studies conducted in routine care settings and reporting original data on guselkumab in adult patients with psoriasis, PsA, or both. Both prospective and retrospective cohort designs were eligible, as were registry analyses and real-world multicenter chart reviews. For psoriasis, eligible studies had to report at least one extractable clinical outcome based on PASI response thresholds, absolute PASI targets, or patient-reported quality-of-life endpoints. For PsA, eligible studies had to report DAPSA-based outcomes or clinically comparable joint-domain measures such as enthesitis indices or pain measures, although only sufficiently repeated outcomes were taken forward to quantitative pooling.

Exclusion criteria were: premarketing randomized trials; reviews, editorials, narrative overviews, and case reports; studies lacking extractable response data; and studies centered on a highly specific selected population when their inclusion would introduce major comparability problems in the pooled analysis. Importantly, specific populations such as elderly patients, patients with malignancy, and cohorts enriched for axial disease were not excluded from the review itself, but could be removed from the primary quantitative synthesis if they represented subcohorts with a high risk of overlap or lack of representativeness.

### 2.4. Study Selection and Overlap Resolution

Study selection was performed in sequential stages: duplicate removal, title screening, abstract screening, and full-text assessment. During full-text assessment, special attention was paid to the possibility of cohort overlap, particularly among serial publications from the same national network, center, or registry. This issue was a major methodological focus because several guselkumab RWE studies represent updated follow-up analyses, subanalyses of the same prospective program, or clinically enriched subcohorts.

The overlap strategy was predefined at the analytical level. Publications judged to derive from the same underlying cohort were not allowed to contribute concurrently to the same pooled estimate for the same outcome and time window. For example, PERSIST-related publications from the Gerdes group were treated as a single cohort family, and only the most appropriate efficacy dataset was retained for the primary pool. Likewise, smaller or earlier Valenti [16] and Mastorino [26] publications were not allowed to compete with the larger or more mature cohorts when patient duplication was probable. Subcohorts such as elderly-only psoriasis cohorts and axial- enriched PsA cohorts were preserved for qualitative synthesis and discussion but not forced into the main pooled analysis.

### 2.5. Data Extraction and Denominator Handling

Data extraction included publication year, country, design, sample size, follow-up schedule, disease indication, treatment history context, and study outcomes. For the quantitative synthesis, the unit of analysis was the study-level proportion of patients achieving a predefined endpoint within a prespecified time window. When exact numerators and denominators were explicitly reported in tables or the main text, those counts were used directly. This was the case for key anchor studies such as the large multicenter Valenti 2025 [16] psoriasis cohort.

When only percentages were reported in the accessible full text or abstract, counts were reconstructed by multiplying the published percentage by the reported sample size and rounding to the nearest whole number. To maintain methodological transparency, each study-level record in the final dataset was flagged according to the denominator type used by the source study: intention-to-treat (ITT), evaluable population, or completer population. This distinction was considered essential because some long-term observational reports calculated response rates only among patients still receiving treatment at the corresponding visit.

### 2.6. Outcomes and Time Windows

For psoriasis, the primary pooled outcomes were PASI 90 and PASI 100. PASI 75 was considered a supportive secondary endpoint because of its continued relevance to treatment response benchmarking in observational studies. Although other outcomes such as absolute PASI ≤ 2, DLQI 0/1, and investigator global assessments were extracted where available, they were less uniformly reported and were therefore used primarily in qualitative interpretation rather than the main pooled display.

For PsA, the primary pooled outcome was DAPSA < 14, reflecting low disease activity. Other PsA outcomes, including DAPSA remission, pain visual analogue scale, LEI, and composite indices, were extracted qualitatively but not meta-analyzed unless reporting was sufficiently repetitive and methodologically compatible.

Because real-world studies reported follow-up at slightly different nominal visits, clinically coherent windows were defined a priori. For psoriasis, windows were 12–16 weeks, 20–28 weeks, 36–60 weeks, and an exploratory long-term window of ≥96 weeks. For PsA, the main windows were 20–28 weeks and 48–60 weeks. These windows were chosen to preserve comparability while avoiding unnecessary loss of data from studies reporting week 24, 36, 48, 52, or 60 outcomes.

### 2.7. Risk of Bias Assessment

Risk of bias was assessed according to study design. Cohort studies were appraised using the Newcastle-Ottawa Scale (NOS), while before-after cohorts without a control arm were judged with the NIH quality assessment framework for before-after studies [27]. The final appraisal focused on selection, outcome measurement, comparability/confounding, follow-up completeness, and analytical transparency. Given the consistent single-arm nature of the evidence base, most studies were ultimately categorized as moderate quality rather than low risk of bias, even when conduct was otherwise robust.

### 2.8. Statistical Analysis

The meta-analysis was conducted as a random-effects synthesis of proportions using logit- transformed event rates. For each study, the observed event proportion p = x/n was transformed as logit(p) = log[p/(1 − p)]. A continuity correction was applied for cells with zero events or events in all participants to avoid undefined logits. This approach was used to handle extreme proportions and allow inclusion of studies with boundary event rates in the pooled analysis. In addition, sensitivity analyses were used to explore the robustness of selected pooled estimates, including leave-one-out analysis for the psoriasis PASI 90 maintenance window. Within-study variances were calculated using the inverse binomial approximation on the transformed scale. Between-study variance was estimated with the DerSimonian-Laird method, and pooled estimates were back-transformed to the original proportion scale for reporting. The DerSimonian–Laird estimator was selected due to its widespread use and interpretability in meta-analyses of proportions. However, it is acknowledged that this method may underestimate uncertainty in the presence of a small number of studies or substantial heterogeneity. Alternative approaches, such as restricted maximum likelihood (REML) with Hartung–Knapp adjustment, may provide more conservative estimates and are discussed as a methodological limitation.

Heterogeneity was quantified using Cochran’s Q, tau-squared (tau^2^), and I-squared (I^2^). Given the relatively small number of studies in several pools and the uneven sample sizes of contributing cohorts, heterogeneity metrics were interpreted cautiously rather than mechanistically. All analyses were implemented in Python 3 using custom scripts with pandas, numpy, matplotlib, and python-docx for downstream reporting. The final study-level dataset, meta-summary table, and figure files were generated from the same analysis pipeline to maintain internal consistency across outputs.

Key methodological decisions, including the definition of primary outcomes (PASI 90/100 and DAPSA < 14), time-window grouping, cohort overlap handling rules, and statistical modeling strategy, were specified prior to data extraction and quantitative synthesis.

## 3. Results

### 3.1. Study Selection

**Figure 1:**
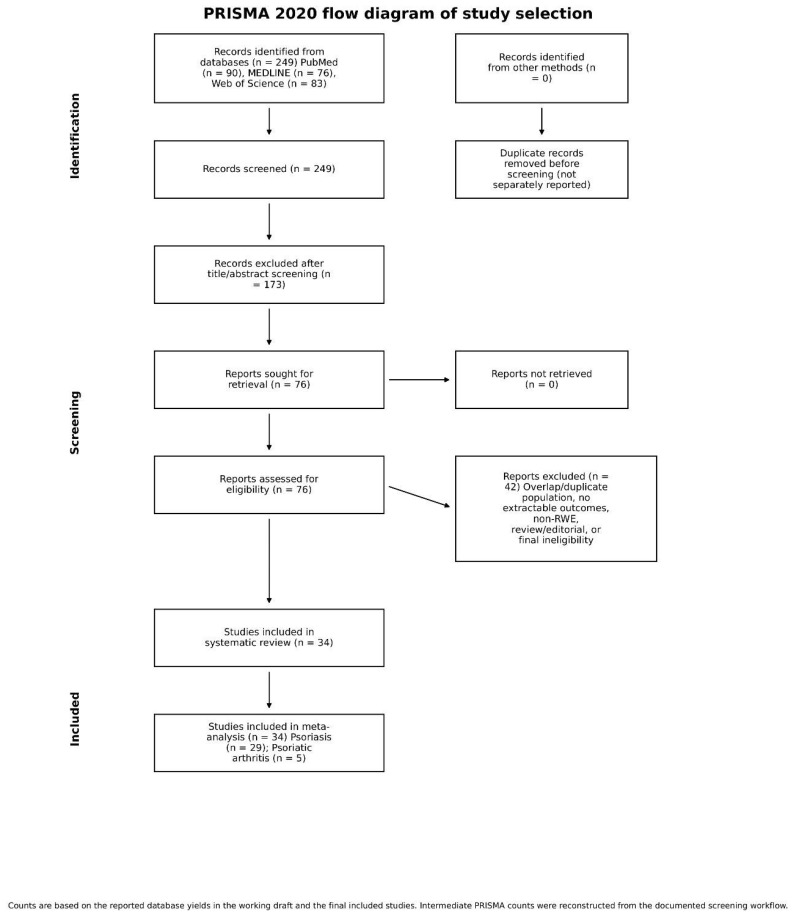
PRISMA 2020 flow diagram of study identification, screening, eligibility assessment, and inclusion.

The PRISMA flow diagram is shown in Figure 1. The database search yielded 249 records. After duplicate removal, 99 records were screened by title and abstract. Twenty-seven records were excluded during title screening and 24 after abstract review. Forty-eight full-text articles were evaluated in detail, and 14 were excluded after full-text assessment because they were pivotal premarketing trials, non-real-world publications, studies without extractable endpoints, or highly selected cohorts considered unsuitable for the main pooled synthesis. Thirty-four studies were included in the final review, of which 29 focused on psoriasis and 5 on PsA.

### 3.2. Characteristics of the Pooled Studies

Summarized the main characteristics of the studies that contributed directly to the primary quantitative syntheses (Table 1). Most psoriasis studies were retrospective or prospective observational cohorts conducted in Europe, particularly Italy and Germany. The largest psoriasis dataset was Valenti 2025 [16], a multicenter Italian cohort with 1413 patients with cutaneous psoriasis available for the pooled analyses. In PsA, the quantitative synthesis was based mainly on a multicenter.

Italian prospective cohort (Ruscitti 2024 [17]), an early-PsA real-world cohort (Pantano 2022 [18]), and the Italian registry-based GISEA analysis (Atzeni 2025 [19]).

### 3.3. Risk of Bias Assessment

The overall methodological quality of the included studies was moderate. No pooled study was a randomized real-world comparative trial, and most lacked a concurrent control group. The main bias domains were selection bias, uncontrolled confounding, denominator instability at later follow-up visits, and potential selective outcome reporting. Table 2 shows the pragmatic quality assessment used for the pooled studies. Additional qualitative concern came from potential cohort overlap among serial publications from the same networks. This issue was not captured fully by the formal tools and was therefore handled analytically through the overlap-resolution procedure described in the Methods.

### 3.4. Quantitative Synthesis: Psoriasis

#### 3.4.1. PASI 90

**Figure 2:**
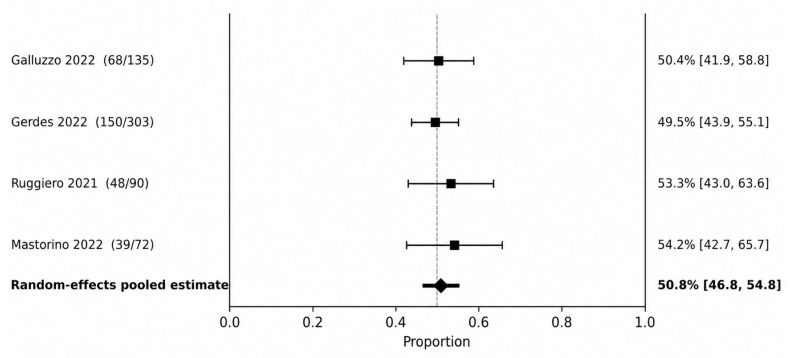
PASI 90 at 12–16 weeks. Random-effects meta-analysis of study-level response proportions [10,12,26,28].

**Figure 3:**
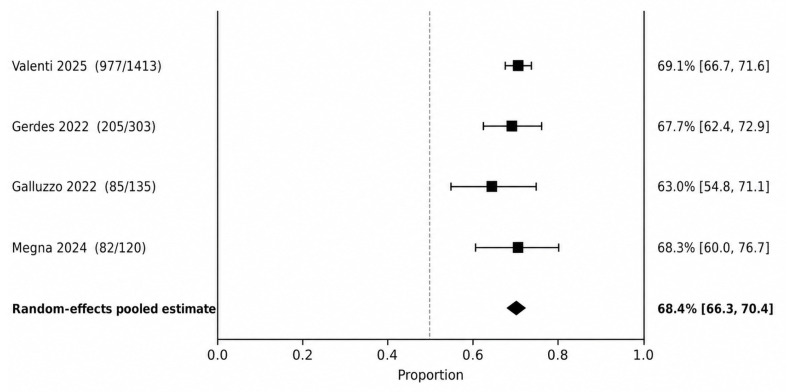
PASI 90 at 20–28 weeks. Random-effects meta-analysis of study-level response proportions [10,12,16,29].

PASI 90 was the most consistently reported psoriasis endpoint and therefore served as the primary efficacy outcome for the cutaneous disease synthesis. At 12–16 weeks (Figure 2), the pooled PASI 90 response rate was 50.8% (95% CI 46.8–54.8), with no statistical heterogeneity detected (I^2^ = 0.0%). At 20–28 weeks (Figure 3), the pooled PASI 90 response increased to 68.4% (95% CI 66.3–70.4; I^2^ = 0.0%).

**Figure 4:**
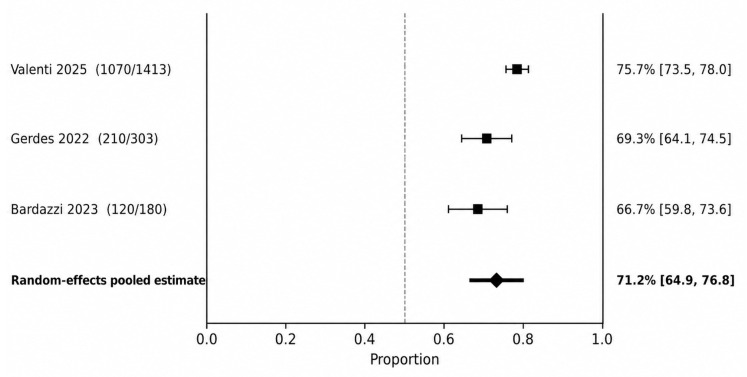
PASI 90 at 36–60 weeks. Random-effects meta-analysis of study-level response proportions [10,14,16].

At 36–60 weeks (Figure 4), the pooled PASI 90 response was 71.2% (95% CI 64.9–76.8), although heterogeneity increased substantially (I^2^ = 81.0%). This higher heterogeneity was clinically plausible and likely reflected differences in study design, denominator choice at maintenance time points, and cohort composition. In the exploratory ≥96-week window, one large study reported a PASI 90 rate of 77.1% (95% CI 74.9–79.3).

To further explore the observed heterogeneity, a leave-one-out sensitivity analysis was performed. Exclusion of the large Valenti 2025 [16] cohort substantially reduced heterogeneity, indicating that this study was a major contributor to between-study variance. However, the pooled effect size remained directionally consistent, supporting the robustness of the overall estimate (Supplementary Table S2).

#### 3.4.2. PASI 100 and PASI 75

**Figure 5:**
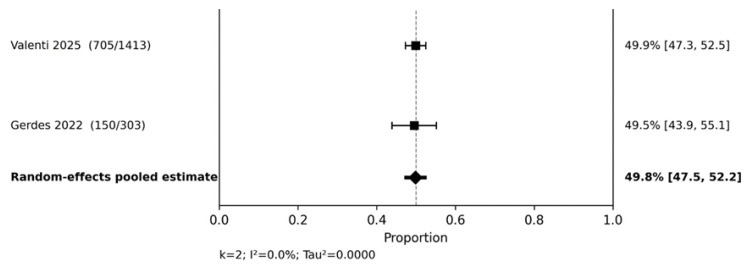
PASI 100 at 20–28 weeks. Random-effects meta-analysis of study-level response proportions [10,16].

Complete skin clearance (PASI 100) was reported sufficiently often for two pooled analyses. At 20–28 weeks (Figure 5), the pooled PASI 100 response was 49.8% (95% CI 47.5–52.2; I^2^ = 0.0%). At 36–60 weeks, one large study reported a PASI 100 response of 49.7% (95% CI 47.1–52.3). Although fewer studies were available than for PASI 90, these findings support the ability of guselkumab to achieve and maintain deep skin clearance in routine practice.

PASI 75 was available for the early psoriasis window, with a pooled response rate of 76.3% (95% CI 72.0–80.0; I^2^ = 0.0%). This higher early pooled response relative to PASI 90 was directionally consistent with expectations and supported the internal coherence of the pooled dataset.

#### 3.4.3. Qualitative Synthesis of Psoriasis Outcomes and Safety

Beyond the pooled endpoints, the qualitative synthesis indicated consistent improvement in absolute PASI targets, quality-of-life measures, and treatment persistence (Table 3). The large Valenti 2025 multicenter cohort showed sustained PASI 90, PASI 100, and absolute PASI ≤ 2 responses through 260 weeks [16]. PERSIST publications from Germany similarly documented clinically meaningful improvements in both effectiveness and health-related quality of life [10,11]. Additional multicenter and single-country cohorts broadly aligned with these findings, despite differences in baseline biologic exposure and disease duration [12,13,14,15,21].

Safety reporting in psoriasis studies was not sufficiently homogeneous for a formal pooled meta-analysis. Nevertheless, the overall pattern was reassuring. Across the extracted safety table, the most frequently reported adverse events were upper respiratory infections, rash, headache, pruritus, and mild gastrointestinal symptoms. Event reporting was usually sparse, and serious adverse events were rare. New safety signals were not identified in long-term cohorts, including in analyses focused on patients with chronic infections, previous cancer, or advanced age [21,22,23].

### 3.5. Quantitative Synthesis: Psoriatic Arthritis

#### 3.5.1. DAPSA < 14

**Figure 6:**
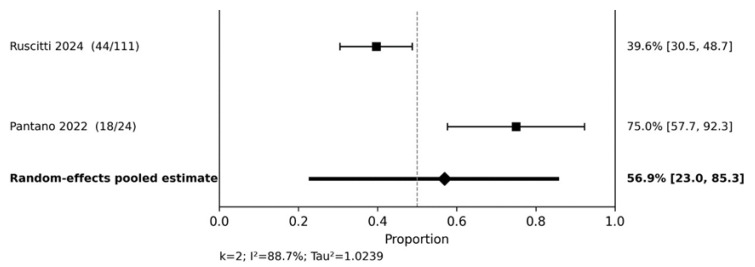
DAPSA < 14 at 20–28 weeks. Random-effects meta-analysis of study-level response proportions [17,30].

The PsA quantitative synthesis focused on DAPSA <14, representing low disease activity. At 20–28 weeks (Figure 6), the pooled DAPSA <14 response was 56.9% (95% CI 23.0–85.3), with high heterogeneity (I^2^ = 88.7%). The wide confidence interval and high heterogeneity reflected the limited number of studies and marked between-study variability, and this estimate should therefore be interpreted as supportive rather than definitive.

**Figure 7:**
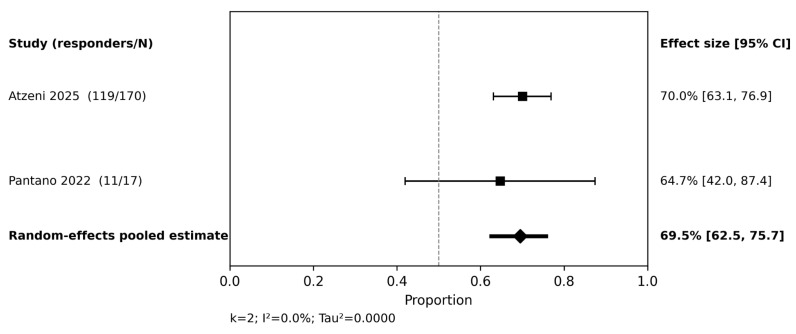
DAPSA < 14 at 48–60 weeks. Random-effects meta-analysis of study-level response proportions [19,30].

At 48–60 weeks (Figure 7), the pooled DAPSA <14 response was 69.5% (95% CI 62.5–75.7; I^2^ = 0.0%). Although statistical heterogeneity was low at this later window, the result still requires caution because denominator definitions differed across studies. The convergent direction of benefit nevertheless supports the maintenance of meaningful joint disease control with continued guselkumab treatment.

#### 3.5.2. Qualitative Synthesis of Additional PsA Outcomes

Qualitative review of the PsA literature suggested improvement across multiple domains beyond the pooled DAPSA endpoint. Atzeni 2025 documented favorable 12-month effectiveness, safety, and retention in the GISEA cohort, while Ruscitti 2024 showed short-term effectiveness and meaningful retention in a prospective multicenter setting [17,19]. Pantano 2022 [18] provided early evidence of benefit in a small real-life early-PsA cohort, including peripheral and axial manifestations.

Studies focusing on specific phenotypes, such as suggestive axial involvement, were retained for narrative context but not pooled in the primary PsA analysis because of the risk of phenotype- driven heterogeneity. This was the case for Foti 2025 and other enriched subcohorts [23]. Similarly, registry studies such as CorEvitas provided valuable external support for effectiveness and persistence in routine care but were not forced into the pooled estimates when outcome harmonization was insufficient [20]. All data was listed at Table 4.

### 3.6. Cohort Overlap Decisions and Analytical Consequences

Supplementary Table S1 summarizes the principal overlap decisions that shaped the final pooled dataset. These decisions were not cosmetic. They changed which studies entered the primary pools and were essential to avoid double-counting. Hoffmann 2021 [30] was retained for narrative health- related quality-of-life context but not pooled concurrently with the 2022 PERSIST efficacy report. Gargiulo 2024 [31] was superseded by the larger Valenti 2025 [16] dataset in the primary psoriasis pools. Mastorino 2022 [26] was excluded from the primary pool because of substantial concern regarding partial duplication with other Turin-series guselkumab cohorts. An elderly-only Ruggiero subcohort was retained for subgroup narrative rather than the main psoriasis synthesis. In PsA, Foti 2025 [23] was classified as a sensitivity or narrative cohort because its axial-enriched phenotype was not judged representative of the pooled general-PsA analysis (Table 5).

## 4. Discussion

This systematic review and meta-analysis provides a focused synthesis of RWE on guselkumab in psoriasis and PsA while explicitly addressing one of the major limitations of the field: serial publication of partially overlapping cohorts. The principal findings are that guselkumab achieves rapid and clinically meaningful skin responses in routine care, maintains those responses over time, and provides clinically relevant improvement in PsA, with a safety profile that remains consistent with the broader development program.

The psoriasis results are notable for both magnitude and temporal durability. In the early window, the pooled PASI 90 response of 50.8% should be viewed as a pragmatic real-world estimate rather than a lower-quality analogue of trial efficacy. By 20–28 weeks, the pooled PASI 90 rate rose to 68.4%, approaching the efficacy range reported in VOYAGE 1 and VOYAGE 2 under trial conditions [5,6]. This convergence is important because real-world cohorts typically include patients with prior biologic exposure, greater comorbidity burden, more variable adherence, and less standardized outcome assessment. In that context, a pooled PASI 90 around two thirds of patients is clinically persuasive.

Deep response was also maintained in the real-world setting. The pooled PASI 100 estimate of 49.8% at 20–28 weeks and the nearly identical 36–60-week estimate support the view that guselkumab is capable not only of achieving high-level skin clearance but also of maintaining it. The apparent plateau in PASI 100 from intermediate to maintenance windows is clinically plausible and likely reflects both the biology of deep clearance and denominator effects at later observational follow-up. The exploratory ≥96-week PASI 90 estimate from Valenti 2025 further reinforces that long-term efficacy is not merely transient [16].

A particularly relevant methodological point is that heterogeneity behaved differently across time windows. At 12–16 and 20–28 weeks, statistical heterogeneity for psoriasis pools was low. This does not necessarily imply the absence of true clinical heterogeneity. With only a few studies per pool and one very large multicenter cohort contributing substantial weight, I^2^ can underestimate between-study variability. By contrast, the 36–60-week PASI 90 analysis showed high heterogeneity (I^2^ = 81.0%), which is more consistent with the underlying clinical reality. Maintenance analyses are especially sensitive to denominator definitions, treatment persistence, and loss-to-follow-up structures. Some cohorts report outcomes on the full baseline population, whereas others use evaluable or completer populations at later visits. This difference alone can materially influence observed response proportions. Importantly, maintenance response rates in observational studies may therefore reflect not only biological durability of treatment effect, but also survivor bias, treatment persistence, and selective retention of responders over time.

The PsA results should be interpreted as clinically encouraging but methodologically more fragile than the psoriasis findings. The early pooled DAPSA <14 response of 56.9% was accompanied by very wide uncertainty (95% CI 23.0–85.3%), reflecting the small number of contributing studies and substantial between-study heterogeneity. Additional sources of variability likely include differences in prior biologic exposure, disease duration, baseline articular burden, phenotype distribution, and denominator definitions across cohorts. These factors are particularly relevant in PsA, where smaller sample sizes and multidomain disease expression can amplify between-study heterogeneity. This wide confidence interval indicates limited precision and underscores that this estimate should be considered exploratory rather than definitive.

Real-world PsA cohorts are typically smaller, more heterogeneous, and differ in biologic history, disease duration, and domain emphasis. Moreover, DAPSA represents only one dimension of PsA disease activity, and improvements in other domains may not be fully captured by this measure [17,18,19,20].

Our findings align with and extend the pivotal trial evidence. VOYAGE 1 and VOYAGE 2 established the efficacy of guselkumab in psoriasis, showing superiority over placebo and adalimumab [5,6]. DISCOVER-1, DISCOVER-2, and COSMOS similarly demonstrated benefit across musculoskeletal and skin domains in PsA, including TNF inhibitor-inadequate responders [7,8,9]. The present study does not claim direct equivalence between RWE and trial outcomes; rather, it shows that the real-world trajectory of benefit is directionally consistent with the trial program and remains substantial even in more complex patient populations.

Safety data merit a deliberately cautious interpretation. We did not force a formal pooled meta- analysis of adverse events because the safety literature was too heterogeneous in event definitions, ascertainment windows, and reporting thresholds. Some studies provided percentages for selected events only, others reported isolated adverse-event narratives, and only a subset clearly distinguished any adverse event from serious adverse events or treatment discontinuations due to toxicity. Nevertheless, the qualitative safety pattern was coherent across studies. However, the absence of a pooled safety signal should not be interpreted as equivalence with randomized trial safety profiles, but rather as a reflection of the limitations of adverse event ascertainment, reporting thresholds, and heterogeneity in real-world studies. The most commonly recurring events were upper respiratory infections, mild rash, headache, pruritus, and gastrointestinal symptoms. Serious adverse events were uncommon, and no new safety signal emerged from long-term practice-based cohorts or studies focused on vulnerable populations such as patients with comorbidity, prior malignancy, or advanced age [21,22,23].

The handling of cohort overlap is, in our view, one of the main strengths of this manuscript. The guselkumab RWE literature contains several families of publications that can easily be double-counted if not mapped carefully. By deciding in advance which dataset would represent each cohort family within each pooled outcome and time window, we preserved the interpretability of the pooled estimates. This is especially relevant for readers who may otherwise compare pooled proportions across reviews without recognizing that serial publications from the same underlying patients can inflate precision artificially.

The study also has clear limitations. First, all included studies were observational, and most lacked a concurrent comparator. Residual confounding and selection bias are therefore unavoidable. Second, some event counts had to be reconstructed from published percentages when exact numerators were not available in the accessible report version. Although this process was performed transparently and flagged in the dataset, it may introduce minor imprecision due to rounding, particularly in smaller cohorts, and could slightly affect pooled estimates. Third, denominator definitions varied across studies, particularly in later follow-up windows. This likely contributed to the heterogeneity seen in maintenance analyses and may also affect apparent response stability over time. Fourth, the PsA evidence base remains comparatively small, limiting the number of outcomes that can be pooled robustly. Finally, because this review was not prospectively registered, there is an additional reporting limitation that must be acknowledged. Formal assessment of reporting bias (e.g., funnel plots) and certainty of evidence (e.g., GRADE) was not performed. This decision was driven by the small number of studies in several pooled analyses, the use of proportion-based outcomes, and substantial between-study heterogeneity, which limit the interpretability of funnel plot asymmetry and the applicability of GRADE frameworks. Nevertheless, the potential for reporting bias should be acknowledged. RWE studies may overestimate treatment effectiveness due to selective publication of positive results, incomplete outcome reporting, and attrition-related biases. From a clinical perspective, these findings support the use of guselkumab in routine practice not only in trial-like populations, but also in more heterogeneous patients encountered in everyday dermatology and rheumatology care.

Despite these limitations, the clinical implications are straightforward. Guselkumab appears highly effective in real-world psoriasis and meaningfully effective in real-world PsA, with durability extending into the maintenance phase and a safety profile that remains favorable. The data support its use not only in selected trial-like populations but also across broader everyday practice settings that include biologic-experienced patients and those with multimorbidity. Future work should prioritize standardized outcome reporting, clearer safety denominators, prospective comparative RWE, and better harmonization of psoriasis and PsA domain measures to enable more informative pooled analyses.

## 5. Conclusions

This systematic review and meta-analysis support guselkumab as an effective real-world treatment for moderate-to-severe psoriasis and as a clinically useful option for PsA. In psoriasis, the pooled results indicate rapid improvement by 12–16 weeks, strong PASI 90 responses by 20–28 weeks, and sustained maintenance responses through 36–60 weeks and beyond. In PsA, pooled DAPSA < 14 responses suggest that continued guselkumab treatment can achieve and maintain low disease activity for a meaningful proportion of patients.

Methodological caveats remain important. The evidence base is observational, several pools include only a small number of studies, safety reporting is heterogeneous, and later time points may depend on evaluable or completer denominators. Nonetheless, the direction, magnitude, and consistency of benefit across the available evidence strongly support the use of guselkumab in routine practice. The present manuscript also underscores the importance of explicit overlap resolution in future RWE syntheses.

## Figures and Tables

**Table 1:** Characteristics of the studies contributing to the primary pooled analyses.

Study	Country	Design	Indication	N	Follow-Up	Main Outcomes	Role in Synthesis
Gerdes 2022 [10]	Germany	Prospective multicenter cohort	Psoriasis	303	12, 28, 52 weeks	PASI 90/100, safety	Primary pool; PERSIST anchor
Galluzzo 2022 [12]	Italy	Retrospective cohort	Psoriasis	135	12 and 52 weeks	PASI 75/90	Primary pool
Ruggiero 2021 [28]	Italy	Retrospective cohort	Psoriasis	90	16 weeks	PASI 90	Primary pool
Mastorino 2022 [26]	Italy	Prospective cohort	Psoriasis	72	16 weeks	PASI 90	Primary pool (early window only)
Valenti 2025 [16]	Italy	Multicenter retrospective cohort	Psoriasis	1413	16, 28, 52, 104, 156, 204, 260 weeks	PASI 90/100, PASI ≤ 2, safety	Primary pool; exact numerators verified
Megna 2024 [29]	Italy	Retrospective cohort	Psoriasis	120	24 weeks	PASI 90	Primary pool
Bardazzi 2022/2023 [14]	Italy	Multicenter retrospective cohort	Psoriasis	180	36–60 weeks	PASI 90, safety	Primary pool (maintenance)
Ruscitti 2024 [17]	Italy	Prospective multicenter cohort	PsA	111	24 weeks, 18-month retention	DAPSA < 14, DRR	Primary PsA pool
Pantano 2022 [18]	Italy	Prospective cohort	PsA	24	24 and 52 weeks	DAPSA low activity/remission	Primary PsA pool; completer denominator at 12 months
Atzeni 2025 [19]	Italy	Registry multicenter cohort	PsA	170	6 and 12 months	DAPSA < 14, MDA, retention	Primary PsA pool with evaluable denominator flag

**Table 2:** Risk of bias assessment of studies contributing to the primary quantitative synthesis.

Study	Design	Tool	Score	Overall_Judgement	Key_Comment
Gerdes 2022 [10]	Prospective cohort	NOS	7/9	Moderate	No control group; strong follow-up, prospective multicenter design
Galluzzo 2022 [12]	Retrospective cohort	NOS	6/9	Moderate	Observational design, single- arm
Ruggiero 2021 [28]	Retrospective cohort	NOS	6/9	Moderate	Real-world cohort; limited comparability
Mastorino 2022 [26]	Prospective cohort	NOS	6/9	Moderate	Single-center and no comparator
Valenti 2025 [16]	Multicenter cohort	NOS	7/9	Moderate	Large multicenter cohort; selection/confounding remain
Megna 2024 [29]	Retrospective cohort	NOS	6/9	Moderate	Observational design
Bardazzi 2022/2023 [14]	Retrospective cohort	NOS	6/9	Moderate	Observational design
Ruscitti 2024 [17]	Prospective multicenter cohort	NOS	7/9	Moderate	Prospective PsA cohort without control group
Pantano 2022 [18]	Prospective cohort	NIH before-after	Fair	Moderate	Small sample; completer analysis at 12 months
Atzeni 2025 [19]	Registry cohort	NOS	7/9	Moderate	Registry strengths, but evaluable denominator at 12 months

NOS: Newcastle-Ottawa Scale; NIH: National Institutes of Health before-after quality assessment framework.

**Table 3:** Narrative overview of recurrently reported adverse events in the safety extraction table.

Adverse Event Category	Studies Reporting	Observed Range	Interpretive Comment
Upper respiratory infections	8 studies	1.13– 13.0%	Most frequently recurring infectious event; no consistent signal of severe infection burden
Cutaneous rash/eruption	8 studies	0.58– 6.28%	Usually mild and infrequent
Headache	9 studies	sporadically reported	Low absolute frequency across cohorts
Pruritus	5 studies	0.17– 14.16%	Heterogeneous reporting, often mild
Gastrointestinal symptoms	8 studies	0–2.2%	Generally minor and self-limited
Herpes zoster	2 studies	1.2–2.2%	Rare, without a consistent pattern of recurrence

Ranges are descriptive and not pooled because reporting definitions, denominators, and ascertainment windows were heterogeneous across studies.

**Table 4:** Pooled meta-analysis results for primary and supportive outcomes.

Disease	Outcome	Time Window	Studies (k)	Events	Total N	Pooled %	95% CI	I^2^ (%)
Psoriasis	PASI 90	12–16 weeks	4	305	600	50.8	46.8–54.8	0.0
Psoriasis	PASI 90	20–28 weeks	4	1349	1971	68.4	66.3–70.4	0.0
Psoriasis	PASI 90	36–60 weeks	3	1400	1896	71.2	64.9–76.8	81.0
Psoriasis	PASI 90	≥96 weeks	1	1090	1413	77.1	74.9–79.3	0.0
Psoriasis	PASI 100	20–28 weeks	2	855	1716	49.8	47.5–52.2	0.0
Psoriasis	PASI 100	36–60 weeks	1	702	1413	49.7	47.1–52.3	0.0
Psoriasis	PASI 75	12–16 weeks	2	334	438	76.3	72.0–80.0	0.0
PsA	DAPSA <14	20–28 weeks	2	62	135	56.9	23.0–85.3	88.7
PsA	DAPSA <14	48–60 weeks	2	130	187	69.5	62.5–75.7	0.0

Pooled estimates were generated using random-effects meta-analysis of logit-transformed proportions.

**Table 5:** Study-level analytical decisions for cohort overlap and primary-pool inclusion.

Study_Family	Disease	Status	Rationale
Hoffmann 2021 [30]	Psoriasis	Narrative only	Potential overlap with PERSIST/Gerdes 2022 [10]; retained for HRQoL context, not pooled for efficacy
Gerdes 2022 [10]	Psoriasis	Included	Anchor efficacy publication for PERSIST cohort
Gerdes 2025 [11]	Psoriasis	Narrative/long- term context	Avoided concurrent pooling with earlier PERSIST efficacy windows
Gargiulo 2024 [31]	Psoriasis	Sensitivity/narrative	Superseded by larger Valenti 2025 [16] cohort in primary pools
Valenti 2025 [16]	Psoriasis	Included	Primary multicenter long-term cohort; exact counts verified for key PASI outcomes
Mastorino 2022 [26]	Psoriasis	Excluded from primary pool	High risk of partial duplication with other Turin-series guselkumab cohorts
Ruggiero 2021 elderly subcohort [28]	Psoriasis	Excluded from primary pool	Special elderly subcohort; retained for subgroup narrative only
Foti 2025 [23]	PsA	Narrative/sensitivity	Axial-enriched PsA cohort; not pooled in general PsA primary analysis
Atzeni 2025 [19]	PsA	Included with flag	Primary PsA registry cohort; denominator treated as evaluable at 12 months

## Data Availability

No new data were created or analyzed in this study. Data sharing is not applicable to this article.

## Supplementary Materials

The following supporting information can be downloaded at: https://www.mdpi.com/article/10.3390/jcm15103692/s1, Table S1: PRISMA 2020 CHECKLIST; Table S2: Leave-one-out analysis for PASI 90 (36–60 weeks). File-S1: Full-Search-Strategy.

## References

[1] Page M.J., McKenzie J.E., Bossuyt P.M., Boutron I., Hoffmann T.C., Mulrow C.D., Shamseer L., Tetzlaff J.M., Akl E.A., Brennan S.E. (2021). The PRISMA 2020 statement: An updated guideline for reporting systematic reviews. BMJ.

[2] Griffiths C.E.M., Armstrong A.W., Gudjonsson J.E., Barker J.N.W.N. (2021). Psoriasis. Lancet.

[3] Ogdie A., Weiss P. (2015). The epidemiology of psoriatic arthritis. Rheum. Dis. Clin. N. Am..

[4] Jeon C., Sekhon S., Yan D., Afifi L., Nakamura M., Bhutani T. (2017). Monoclonal antibodies inhibiting IL-12, -23, and -17 for the treatment of psoriasis. Hum. Vaccin. Immunother..

[5] Blauvelt A., Papp K.A., Griffiths C.E., Randazzo B., Wasfi Y., Shen Y.-K., Li S., Kimball A.B. (2017). Efficacy and safety of guselkumab, an anti-interleukin-23 monoclonal antibody, compared with adalimumab for the continuous treatment of patients with moderate-to-severe psoriasis: Results from the phase III, double-blinded, placebo- and active comparator-controlled VOYAGE 1 trial. J. Am. Acad. Dermatol..

[6] Reich K., Armstrong A.W., Foley P., Song M., Wasfi Y., Randazzo B., Li S., Shen Y.-K., Gordon K.B. (2017). Efficacy and safety of guselkumab, an anti-interleukin-23 monoclonal antibody, compared with placebo and adalimumab for the treatment of patients with moderate-to-severe psoriasis with randomized withdrawal and retreatment: Results from the phase III, double-blind, placebo- and active comparator-controlled VOYAGE 2 trial. J. Am. Acad. Dermatol..

[7] Deodhar A., Helliwell P.S., Boehncke W.-H., Kollmeier A.P., Hsia E.C., ASubramanian R., Xu X.L., Sheng S., Agarwal P., Zhou B. (2020). Guselkumab in patients with active psoriatic arthritis who were biologic-naive or had previously received TNFalpha inhibitor treatment (DISCOVER-1): A double-blind, randomised, placebo- controlled phase 3 trial. Lancet.

[8] Mease P.J., Rahman P., Gottlieb A.B., Kollmeier A.P., Hsia E.C., Xu X.L., Sheng S., Agarwal P., Zhou B., Zhuang Y. (2020). Guselkumab in biologic-naive patients with active psoriatic arthritis (DISCOVER-2): A double-blind, randomised, placebo-controlled phase 3 trial. Lancet.

[9] Coates L.C., Gossec L., Theander E., Bergmans P., Neuhold M., Karyekar C.S., Schett G., McInnes I.B., Mease P.J., Nash P. (2022). Efficacy and safety of guselkumab in patients with active psoriatic arthritis and inadequate response to TNF inhibitors: Results through one year of the phase IIIb COSMOS study. Ann. Rheum. Dis..

[10] Gerdes S., Mrowietz U., Franke J., Korge B., Philipp S., Kokolakis G., Kirsten N., Thaci D., Reich K., Augustin M. (2022). Real-world evidence from the non-interventional, long-term, German mu ticentre PERSIST study of guselkumab in patients with moderate-to-severe psoriasis. J. Eur. Acad. Dermatol. Venereol..

[11] Gerdes S., Mrowietz U., von Kiedrowski R., Franke J., Hoffmann M., Kokolakis G., Kirsten N., Thaci D., Reich K., Augustin M. (2023). Effectiveness, survival and safety of guselkumab with patient-reported quality-of-life outcomes in routine care. J. Eur. Acad. Dermatol. Venereol..

[12] Galluzzo M., D’Adamio S., Silvaggio D., Tofani L., Bianchi L. (2022). Real-world outcomes in patients with moderate-to-severe plaque psoriasis treated with guselkumab for up to 12 months. Expert Opin. Biol. Ther..

[13] Galluzzo M., Talamonti M., Narcisi A., D’Adamio S., Bianchi L. (2023). Guselkumab for treatment of moderate-to-severe chronic plaque psoriasis in routine practice: Real-life effectiveness and drug-survival for up to 148 weeks. Expert Opin. Biol. Ther..

[14] Bardazzi F., Balestri R., Loi C., Bianchi F., Balestri A., Patrizi A. (2022). Guselkumab for the treatment of psoriasis: A 60-week real-life multicenter experience. Expert Opin. Biol. Ther..

[15] Malkonen T., Snellman E., Pukkala E., Reunala T., Huilaja L., Tasanen K., Hannula-Jouppi K., Heikkilä H., Karppinen T., Kokkonen N. (2022). Guselkumab treatment outcomes and persistence in a nationwide Finnish real-life setting. Acta Derm.-Venereol..

[16] Valenti M., Ibba L., Di Giulio S., Dapavo P., Malagoli P., Marzano A.V., Loconsole F., Burlando M., Balato A., Dini V. (2025). Guselkumab retention, effectiveness, and safety in psoriasis: A 260-week multicenter real-world study. Dermatol. Ther..

[17] Ruscitti P., Cataldi G., Gentile M., Dionisi A., Volpe P., Finucci A., Verardi L., Di Muzio C., Italiano N., Celletti E. (2024). The evaluation of effectiveness and safety of guselkumab in patients with psoriatic arthritis in a real-life multicentre cohort. J. Clin. Med..

[18] Pantano I., Mauro D., Romano F., Gambardella A., Valenti M., Simone D., Iacono D., Costanzo A., Argenziano G., Ciccia F. (2022). Real-life efficacy of guselkumab in patients with early psoriatic arthritis. Rheumatology.

[19] Atzeni F., Rotondo C., Siragusano C., Corrado A., Cauli A., Caporali R., Chimenti M.S., Conti F., Picerno V., Gremese E. (2025). Italian multicenter real-world study on the twelve-month effectiveness, safety, and retention rate of guselkumab in psoriatic arthritis patients. J. Clin. Med..

[20] Mease P.J., Hsia E.C., Kimball A.B., Goyal K., Banerjee S., Han C., Wang Y., Lehman T.J.A., Curtis J.R., Greenberg J.D. (2023). Real-world data from the CorEvitas Psoriatic Arthritis/Spondyloarthritis Registry on guselkumab effectiveness and persistence. Rheumatol. Ther..

[21] Mortato E., Talamonti M., Marcelli L., Megna M., Raimondo A., Caldarola G., Bernardini N., Balato A., Campanati A., Esposito M. (2025). Long-term real-world effectiveness and drug survival of guselkumab in patients with psoriasis: A 5-year retrospective study. Psoriasis.

[22] Fratton Z., Chimenti S., Chiricozzi A., Saraceno R., Bianchi L., Talamonti M., Dattola A., Narcisi A., Perricone C., Gisondi P. (2025). Real-world experience of guselkumab in the elderly population with psoriasis. Dermatol. Ther..

[23] Foti R., Cacciapaglia F., Rotondo C., D’Angelo S., Lubrano E., Marchesoni A., Favalli E.G., Becciolini A., Iannone F., Olivieri I. (2025). Guselkumab in psoriatic arthritis: Therapeutic impact on axial and peripheral domains in a real-world cohort. J. Clin. Med..

[24] Page M.J., Moher D., Bossuyt P.M., Boutron I., Hoffmann T.C., Mulrow C.D., Shamseer L., Tetzlaff J.M., Akl E.A., Brennan S.E. (2021). PRISMA 2020 explanation and elaboration: Updated guidance and exemplars for reporting systematic reviews. BMJ.

[25] Wells G.A., Shea B., O’Connell D., Peterson J., Welch V., Losos M., Tugwell P. The Newcastle–Ottawa Scale (NOS) for Assessing the Quality of Nonrandomized Studies in Meta-Analyses. Ottawa Hospital Research Institute. https://www.ohri.ca/programs/clinical_epidemiology/oxford.asp.

[26] Mastorino L., Siliquini N., Avallone G., Zenone M., Ortoncelli M., Quaglino P., Dapavo P., Ribero S. (2022). Guselkumab shows high efficacy and maintenance in the improvement of response until week 48, a real-life study. Dermatol. Ther..

[27] National Heart, Lung, and Blood Institute Study Quality Assessment Tools. https://www.nhlbi.nih.gov/health-topics/study-quality-assessment-tools.

[28] Ruggiero A., Fabbrocini G., Cinelli E., Megna M. (2021). Guselkumab and risankizumab for psoriasis: A 44-week indirect real-life comparison. J. Am. Acad. Dermatol..

[29] Megna M., Ruggiero A., Marasca C., Fabbrocini G. (2024). Real-life effectiveness and safety of guselkumab in patients with moderate-to-severe psoriasis: A 24-week retrospective study. J. Clin. Med..

[30] Hoffmann M., Sticherling M., Korge B., Mortazawi D., Personke Y., Gomez M., Wegner S., Gerdes S. (2021). Psoriasis in routine clinical care: Complete skin clearance rates with guselkumab increase through week 52—Results from the real-life PERSIST study. J. Am. Acad. Dermatol..

[31] Gargiulo L., Ibba L., Cortese A., Toso F., Vignoli C.A., Fiorillo G., Piscazzi F., Valenti M., Costanzo A., Narcisi A. (2024). Real-life Effectiveness and Safety of Guselkumab in Moderate-to-Severe Plaque Psoriasis: A 104-Week Retrospective Single-Center Study. J. Drugs Dermatol..

