# Supplementary material for: Efficacy and Safety of Guselkumab in Real-World Evidence: A Systematic Review and Meta-Analysis
Source: J Clin Med. 2026 May 11;15(10):3692. doi: 10.3390/jcm15103692 (PMC13207209; doi:10.3390/jcm15103692)
Supplement: Supplementary file 1 [file jcm-15-03692-s001.zip › jcm-4274596-supplementary-2.pdf]

Supplementary File S1. Full search strategy (PubMed/MEDLINE)

The following search strategy was used in PubMed/MEDLINE:

("guselkumab"[Title/Abstract] OR guselkumab[MeSH Terms])

AND

("psoriasis"[MeSH Terms] OR psoriasis[Title/Abstract]

OR "psoriatic arthritis"[MeSH Terms] OR "psoriatic arthritis"[Title/Abstract] OR PsA[Title/Abstract])

AND

("real world"[Title/Abstract] OR "real-world"[Title/Abstract]

OR "real life"[Title/Abstract] OR "real-life"[Title/Abstract]

OR observational[Title/Abstract] OR "non-interventional"[Title/Abstract]

OR registry[Title/Abstract])

Filters: Humans, Adults (≥18 years)

The search strategy was adapted as appropriate for MEDLINE and Web of Science according to their specific syntax requirements.

Supplementary Table S2. Leave-one-out analysis for PASI 90 (36–60 weeks)

Study removed	Pooled PASI 90 (%)	I^2 (%)
**None**	71.2	81.0
**Valenti 2025 removed**	~66–68	↓ substantial
**Others removed**	similar	minimal change
